# *MET* and *AKT* Genetic Influence on Facial Emotion Perception

**DOI:** 10.1371/journal.pone.0036143

**Published:** 2012-04-27

**Authors:** Ming-Teng Lin, Kuo-Hao Huang, Chieh-Liang Huang, Yu-Jhen Huang, Guochuan E. Tsai, Hsien-Yuan Lane

**Affiliations:** 1 Institute of Clinical Medical Science, China Medical University, Taichung, Taiwan; 2 Department of Psychiatry, Zhudong Veterans Hospital, Hsinchu, Taiwan; 3 Department of Psychiatry, China Medical University Hospital, Taichung, Taiwan; 4 Department of Psychiatry, Harbor-UCLA Medical Center, Los Angeles Biomedical Research Institute, Torrance, California, United States of America; City of Hope National Medical Center and Beckman Research Institute, United States of America

## Abstract

**Background:**

Facial emotion perception is a major social skill, but its molecular signal pathway remains unclear. The *MET*/*AKT* cascade affects neurodevelopment in general populations and face recognition in patients with autism. This study explores the possible role of *MET*/*AKT* cascade in facial emotion perception.

**Methods:**

One hundred and eighty two unrelated healthy volunteers (82 men and 100 women) were recruited. Four single nucleotide polymorphisms (SNP) of *MET* (rs2237717, rs41735, rs42336, and rs1858830) and *AKT* rs1130233 were genotyped and tested for their effects on facial emotion perception. Facial emotion perception was assessed by the face task of Mayer-Salovey-Caruso Emotional Intelligence Test (MSCEIT). Thorough neurocognitive functions were also assessed.

**Results:**

Regarding *MET* rs2237717, individuals with the CT genotype performed better in facial emotion perception than those with TT (*p* = 0.016 by ANOVA, 0.018 by general linear regression model [GLM] to control for age, gender, and education duration), and showed no difference with those with CC. Carriers with the most common *MET* CGA haplotype (frequency = 50.5%) performed better than non-carriers of CGA in facial emotion perception (*p* = 0.018, df = 1, F = 5.69, p = 0.009 by GLM). In *MET* rs2237717*/AKT* rs1130233 interaction, the C carrier/G carrier group showed better facial emotion perception than those with the TT/AA genotype (*p* = 0.035 by ANOVA, 0.015 by GLM), even when neurocognitive functions were controlled (*p* = 0.046 by GLM).

**Conclusions:**

To our knowledge, this is the first study to suggest that genetic factors can affect performance of facial emotion perception. The findings indicate that *MET* variances and *MET/AKT* interaction may affect facial emotion perception, implicating that the *MET*/*AKT* cascade plays a significant role in facial emotion perception. Further replication studies are needed.

## Introduction

Facial emotion perception is a key element of social function [1], [2], [3]. Patients with schizophrenia have deficits in not only facial emotion expression [4], [5] but also facial emotion perception [6]. Better facial emotion perception is related to better work functioning and independent living in patients with schizophrenia [7]. Unaffected siblings of schizophrenia patients are also impaired in emotion perception [8], [9], [10], implying that deficits in facial emotion perception may transmit in families and serve as a heritable endophenotype of schizophrenia. Facial emotion processing is mainly executed in amygdala, right fusiform gyri, and hippocampus in both schizophrenia patients and healthy individuals [11], [12]. Compared to healthy controls, schizophrenic patients have a hypoactivation of amygdala and hippocampus when processing facial emotions [11], [12]. Relative to healthy subjects without family history of psychosis, healthy offspring of schizophrenia patients also have reduced amygdala activity in response to positive emotional faces [13].

Several studies, focusing on monoamine pathways, suggest that genetic variances can determine activities of relevant brain regions when people perceive facial emotion. Non-depressed adults with short allele of deletion polymorphism in the serotonin transporter-linked polymorphic region (5-HTTLPR) have impaired emotion processing and difficulty disengaging attention from facial stimuli [14]. Healthy subjects carrying G allele of serotonin 1A (5HT1A) C(-1019)G polymorphism have decreased amygdala activity when perceiving fear face [15]. Normal individuals with C/C genotype of 5HT3A C178T polymorphism have greater and faster amygdala activity than C/T heterozygotes when performing facial recognition tasks [16]. Bipolar patients' family members who have Val158 allele of catechol-O-methyltransferase (COMT) valine-to-methionine (Val158Met) polymorphism have greater amygdala activity upon receiving sad facial stimuli [17]. COMT Val158Met polymorphism also affects early phase of facial stimuli processing in children [18].

However, whether genetic factors can affect performance of facial emotion perception remains uncertain. In addition, the signal cascades of facial emotion perception deserve further studies because accumulating evidence suggests that regardless of the underlying genetic complexity, the pathophysiology and endophenotypes of schizophrenia may be determined by a small number of common signaling pathways [19], [20].

*MET*, located at chromosome 7q31, is expressed in amygdala, hippocampus, and cerebral cortex [21], [22], [23]. With 21 exons, *MET* is vital for cerebral and cerebellar development [24], [25], [26], [27] and interneuron migration, which is implicated in pathophysiology of neurodevelopment disorders such as autism and schizophrenia [27], [28], [29]. *MET* is associated with autism [29], [30], [31], [32] and face recognition in autistic patients [33], [34], [35]. Schizophrenia shares common genetic factors and clinical features with autism [36], [37]. Furthermore, Burdick et al found that most common GCAATACA haplotype (47%) from rs38857- rs10215153- rs2237717 - rs2283053- rs41735 - rs41741- rs42336 - rs41750 is less-represented in schizophrenia patients and related with better cognitive performances in the healthy group [37]. Among the 8 SNPs, rs2237717, rs41735, and rs42336 were significantly associated with schizophrenia in two independent case-control comparisons [37]. Moreover, *MET* promoter SNP rs1858830 has been shown to alter gene transcription [29] and be correlated with levels of *MET* protein [38]. In the current study, we focused on these 4 SNPs.

*MET* activates phosphorylation of *AKT*, and this cascade is essential for anti-apoptotic signaling [39] and neuroprotection [40]. The *AKT* gene is also associated with schizophrenia [41], [42], [43]. Haloperidol, a dopamine receptor antagonist, can increase phosphorylation of AKT proteins in brains of mice, implying that *AKT* may be involved in the dopamine neurotransmission pathway [41]. One SNP (rs1130233) of *AKT* can affect amount of AKT protein in lymphocytes and postmortem frontal cortex and hippocampus regions [41]. Subjects with the A allele of *AKT* rs1130233 have impaired executive function, processing speed, trail making, verbal and category fluency and prefrontal cortical structure [44]. AKT is also involved in fear memory learning via the amygdala, a crucial area for emotion perception [45]. However, the role of *AKT* in emotion perception requires elucidation.

This study aimed to testify the genetic effects of the *MET* and *AKT* cascade on facial emotion perception in healthy individuals.

## Methods

This study was approved by the Institutional Review Board of China Medical University Hospital (CMUH), the authors' institutional review board, and performed in accordance with the Declaration of Helsinki. After complete description, all subjects gave written informed consent.

### Subjects

The participants were 182 unrelated Han Chinese healthy individuals (82 men and 100 women) with a mean age of 31.52 (SD = 9.58, range 20–65) years and a mean education level of 14.91 (SD = 2.21, range 6–23 years). All were Han Chinese living in Taiwan. They were free of any axis I or II psychiatric disorders, as determined by a research psychiatrist using the Structured Clinical Interview for DSM-IV [46]. They were also in good physical health, as determined by physical examination, electrocardiogram and laboratory tests including liver, renal and thyroid function tests and urinalysis. After complete description, all subjects gave written informed consent.

### Measurement of facial emotion perception

This study utilized the face task of the emotion perception branch of Mayer-Salovey-Caruso Emotional Intelligence Test (MSCEIT) [47]. MSCEIT is divided into four branches: perceiving emotion (consisting of two tasks: face and picture tasks), facilitating emotion, understanding emotion, and managing emotion [48]. The face task of emotion perception could reflect the ability of being aware of emotional cues before identifying what they mean accurately. In the face task (with four item parcels, each with five responses), participants viewed a series of faces and responded to each one on a five-point scale, indicating the degree to which a specific emotion was present in a face. Patients rated all of the five emotions (happiness, sadness, fear, surprise, and excitement) sequentially [47]. The results were scaled to a standard score based on the weight from the representative adult population (regarding age, gender, and ethnicity) in an extremely large sample of people (5,000) [47]. The reliability and validity of MSCEIT V2.0 have been demonstrated to be favorable in various races [47], [49] including Han-Chinese [50].

### Measurement of cognitive functions

An experienced research psychologist comprehensively assessed cognitive functions to control for their possible effects when we explored genetic effects on facial emotion perception. Among all 182 subjects, 149 also received thorough cognition assessments with a battery of tests, which were the same as or the analogues of tests from Measurement and Treatment Research to Improve Cognition in Schizophrenia (MATRICS) [51], [52]. This battery included 7 domains: (1) speed of processing, consisting of 3 tests: Category Fluency, Trail Marking A, and WAIS-III Digit Symbol-Coding [53], [54]; (2) sustained attention by Continuous Performance Test [55]; (3) working memory, verbal (backward digit span) and nonverbal (WMS-III, Spatial Span) [56]; (4) verbal learning and memory (WMS-III, word listing) [56]; (5) visual learning and memory (WMS-III, visual reproduction) [56]; (6) reasoning and problem solving (WISC-III, Maze) [57], and (7) social cognition, measured by the managing emotions branch of MSCEIT [47]. Mean score of each domain was standardized to a T score with a mean of 50 and a standard deviation of 10. For the domain with more than one test, an overall composite score was calculated by standardizing the sum of T scores [58].

### DNA extraction

Peripheral bloods from schizophrenia subjects were collected in EDTA-tubes. The DNA was isolated by employing the salting-out method [59], and stored in a TE-buffer at 2–8°C. The sample concentrations were measured with a UV/Vis spectrophotometer (Nanodrop ND-1000, Thermo Scientific).

### Genotyping

Four SNPs (rs2237717, rs41735, rs42336, and rs1858830) of the *MET* gene and rs1130233 of the *AKT* gene were selected due to associations with schizophrenia [37], [41], [44] and with performances of neurocognition tests in previous studies [37], [44]. To determine genotyping from venous blood samples, the 5 SNPs expect rs1858830 were amplified via PCR amplification before being subjected to analysis by high resolution melting method (HRM) in ABI 7500 Fast Real-time PCR system (Life technologies).

The sequences of the primers for the *MET* gene were: for rs2237717, sense primer 5′- CCA CGT ACT TCA TCA ATG -3′ and antisense primer 5′- CTT CCT GGC AAT AAA GAG -3′; for rs41735, sense primer 5′- GCT ATT GGA AAA GAA AAG GAT AGA AAC -3′ and antisense primer 5′- CCA TCT GTA GTT GGT AGA ATA TCT CT -3′; for rs42336, sense primer 5′- AGA GAA CTA GAT TAC GTC AGC CAA AGA -3′ and antisense primer 5′- TCT GCC CTG GGG TCA CAT -3′. For rs1130233 of *AKT*, sense primer 5′-GCT GTT CTT CCA CCT GTC -3′ and antisense primer 5′-AGG GCT GAC ACA ATC TCA -3′. Neither a TaqMan Assay-by-Design nor a high resolution melting method (HRM) in ABI 7500 Fast Real-time PCR system (Life technologies) was able to reliably provide rs1858830 genotype from genomic DNA, probably because of ≈85% GC in the region. Therefore the genotype at rs1858830 was determined by direct resequencing.

PCR was performed in a 20-µl volume containing the following: 1× PCR reaction buffer, 1 U FastStart Taq DNA Polymerase (Roche), 0.3 µM of each primer, 1.5 µM SYTO® 9 green fluorescent nucleic acid stain (Life technologies), and 20 ng DNA template, with a 2 mM final MgCl_2_ concentration. The cycling conditions were as follows: denaturation at 95°C for 4 min, followed by 35 cycles of 95°C for 30 s, 58–64°C for 30 s (for *MET* gene: rs2237717 and rs41735 at 58°C; rs42336 at 64°C, and for *AKT* gene: rs1130233 at 63°C), and 72°C for 45 s, followed by one HRM cycle of 95°C for 10 s, 60°C for 1 min, and continuous acquisition to 95°C for 15 s (ramp rate 1%), and subsequently down to 60°C for 15 s. The HRM data were analyzed using the high resolution melting (HRM) software, version 2.0.1. All melting curves deviating from the wild-type curve and appearing as a different color in discrete plots contain a variant potentially. In addition, 24 randomly selected samples including three genotypes of one SNP which identified by HRM were sent to direct sequencing by Mission Biotech CO., LTD, Taiwan.

### Data analysis

All the statistical analyses were performed with the Statistical Package for the Social Sciences (SPSS), version 17.0 for Windows. Deviation of the genotype counts from the Hardy–Weinberg equilibrium was tested by employing a Chi-Square goodness-of-fit test. Chi-Square test was used for gender comparisons between genetic groups. Age, years of education, facial emotion perception and composite cognition between genetic groups was test by ANOVA. Bonferroni correction was used in post-hoc analysis. Linkage disequilibrium structure was examined using Haploview 4.2 [60] with solid spine D′>0.80 (**Figure S1**). *MET* haplotype and diplotype were reconstructed via SAS/GENETICS by implementing the PROC HAPLOTYPE procedure (SAS® 9.1 software). Because *MET* could induce phosphorylation of *AKT* [39], interactions between *MET* and *AKT* may be present in facial emotion perception. Therefore, this study divided subjects into four groups according to the genotype status and function assessment in the previous studies [29], [32], [37], [41], [44] to explore potential interactions between *MET* (rs2237717) and *AKT* (rs1130233) genotypes. The four groups of *MET*/*AKT* were TT/AA, TT/G carrier, C carrier/AA, and C carrier/G carrier.

General linear models (GLM) were utilized to test the main effects of *MET*, *AKT*, and *MET*/*AKT* combination on facial emotion perception, and control variables were age, gender, and years of education as covariates or fixed factors as appropriate. This study utilized partial η2 (eta squared) by SPSS [61] to estimate the effect size in GLM. We also calculated the effect sizes (Cohen's *d*) [62] of the *MET*, *AKT*, and *MET*/*AKT* status to testify whether the combined gene effect was additive or synergistic. Finally, this study utilized G* power 3 [63] to estimate the power and defined the results as statistically significant if the *p*-values were below 0.05 (two-sided).

## Results

All four SNPs of *MET* and *AKT* rs1130233 did not deviate from the Hardy-Weinberg equilibrium. Genotype and allele frequencies of the five SNPs were similar to those of other studies [37], [64], [65] (**Table 1** and **Table S1**). There were high linkage among *MET* rs2237717, rs41735, and rs42336 (D′>0.9); therefore, we constructed haplotype block among the 3 SNPs (**Figure S1**). In this haplotype block, the most common two haplotypes from *MET* rs2237717, rs41735, and rs42336 were CGA and TAG (frequency = 0.505, 0.425, respectively). There were only 2 subjects with CAG haplotype, and their mean [SD] facial emotion perception (88.9[11.0]) was similar to that (84.3[20.5]) of the TAG group; we therefore combined CAG with TAG as the non-CGA group.

**Table 1:** Demographics and facial emotion perception of individuals with different *MET* SNPs, *MET* CGA haplotypes, *AKT* SNP, and *MET*/*AKT* interaction variants.

	Male/Female	Age (SD)	Education (SD)	Facial emotion perception (SD)
***MET*** **- rs2237717**				
CC	19/34	31.4(10.6)	14.4(2.3)	91.9(21.2)
CT	40/48	31.7(9.1)	15.2(1.7)	93.0(18.4)
TT	23/18	31.2(9.4)	14.8(2.9)	83.9(20.9)
*p* *	0.147	0.962	0.097	**0.048**
***MET*** **- rs41735**				
GG	21/33	30.7 (9.8)	14.63(2.2)	91.5(22.0)
GA	45/47	32.2(9.4)	15.20(2.1)	92.3(18.7)
AA	16/20	31.2(9.9)	14.58(2.5)	85.1(20.0)
*p* *	0.500	0.643	0.204	0.175
***MET*** **- rs42336**				
GG	19/18	31.4(9.8)	14.7(3.0)	84.7(20.5)
AG	45/48	31.6(8.9)	15.3(1.8)	92.3(18.9)
AA	18/34	31.5(10.7)	14.4(2.2)	91.9(21.4)
*p* *	0.192	0.993	0.063	0.127
***MET*** **- rs1858830**				
GG	37/42	32.1(10.1)	15.0(2.3)	91.1(20.0)
GC	35/51	31.5(9.4)	14.8(2.2)	90.5(21.1)
CC	10/7	29.1(8.2)	15.1(1.6)	89.5(15.1)
*p* *	0.357	0.498	0.687	0.924
***MET*** **- CGA**				
carrier	58/79	31.6(9.7)	14.9(1.9)	92.7(19.7)
Non carrier†	24/21	31.1(9.2)	15.0(2.9)	84.5(20.1)
*p* *	0.198	0.758	0.865	**0.018**
***AKT*** **- rs1130233**				
AA	23/32	30.3(9.4)	14.6(2.4)	87.0(20.2)
AG	42/49	32.8(9.9)	14.9(2.0)	91.4(22.0)
GG	17/19	30.1(8.7)	15.4(2.4)	94.6(12.9)
*p* *	0.841	0.199	0.199	0.190
***MET-AKT*** ‡				
TT/AA	6/8	31.5(10.0)	13.9(3.1)	80.0 (18.4)
TT/G carrier	17/10	31.1(9.4)	15.3(2.8)	87.0(21.7)
C carrier/AA	17/24	29.9(9.3)	14.8(2.2)	90.1(20.1)
C carrier/G carrier	42/58	32.3(9.8)	15.1(1.7)	93.7(13.2)
*p* *	0.249	0.599	0.279	**0.031**

* Chi-Square test for gender comparison; ANOVA for other items.

† Non carriers of CGA include those with TAG or CAG.

‡ *MET* rs2237717/*AKT* rs1130233.

Demographic characteristics and emotion perception performance by *MET* genotype, *AKT* genotype, *MET* haplotypes, and *MET-AKT* are shown in **Table 1**. Age, gender, and years of education were similar among groups by *MET* genotypes, *AKT* genotypes, or CGA haplotype.

Performances of facial emotion perception were different among 3 genotype groups of *MET* rs2237717 (*p* = 0.048, F = 3.09, df = 2). Bonferroni post hoc analysis revealed this difference was driven from difference between CT and TT (*p* = 0.049; uncorrected *p* = 0.016, power = 0.699). There was a dominant effect of the C allele of *MET* rs2237717, since there was no difference between CT and CC in terms of performance. No differences of facial emotion perception were observed in the other three *MET* SNPs and *AKT* rs113023 (**Table 1**). *MET* CGA haplotype had significant effect on facial emption perception (*p* = 0.018, df = 1, F = 5.69). Subjects with *MET* CGA haplotype also performed better than non-CGA individuals in facial emotion perception by GLM analysis (*p* = 0.009) (**Table 2**).

**Table 2:** General linear regression analyses of effects of the *MET* SNPs, *MET* CGA haplotypes, *AKT* SNP, and *MET*/*AKT* interaction on facial emption perception.*

Genetic variance	Estimated coefficient	Standard error of estimated coefficient	*p*	Power
***MET*** **- rs2237717**				
CC vs. TT	9.987	4.069	**0.015**	0.44
CT vs. TT	8.766	3.683	**0.018**	**0.70**
***MET*** **- rs41735**				
GG vs. AA	6.675	4.213	0.115	0.28
GA vs. AA	5.585	3.883	0.152	0.48
***MET*** **- rs42336**				
GG vs. AA	−8.686	4.219	**0.041**	0.35
GA vs. AA	−2.099	3.450	0.544	0.05
***MET*** **- rs1858830**				
GG vs. CC	2.075	5.303	0.696	0.068
GC vs. CC	2.497	5.268	0.636	0.076
***MET*** **- CGA**				
Carrier vs non-carrier	8.748	3.329	**0.009**	**0.66**
***AKT*** **- rs1130233**				
AA vs. GG	−5.733	4.250	0.179	0.51
AG vs. GG	−2.737	3.887	0.482	0.13
***MET*** **-** ***AKT*** †				
TT/AA‡	−13.626	5.572	**0.015**	**0.81**
TT/G carrier‡	−8.136	4.237	0.056	0.34
C carrier/AA‡	−2.744	3.613	0.449	0.17

* Age, gender, and education years, used as covariates.

† *MET* rs2237717/*AKT* rs1130233.

‡ Compared with C carrier(CT+CC)/G carrier(GA+GG).

Considering the combined effect of *MET* and *AKT* variants, we analyzed *MET* rs2237717 and *AKT* rs1130233 simultaneously. Facial emotion perceptions were different among four combinations of *MET*/*AKT* variants (*p* = 0.031, df = 3, F = 3.03). The Bonferroni post-hoc analysis showed that the difference was driven from the comparison between the individuals who were simultaneously C carriers and G carriers and those with TT/AA (*p* = 0.035, uncorrected *p* = 0.006). In addition, there was nominal difference between TT/AA and C carrier/AA (*p* = 0.049); however it did not survive after Bonferroni correction (*p* = 0.296).

As shown on Table 2, after control for age, gender, and education duration, CC and CT genotypes of *MET* rs2237717increased facial emotion perception by 9.987, 8.766, respectively (*p* = 0.015, 0.018, respectively) when compared with TT genotype. As for *MET* rs42336, AA homozygotes had better performance than those with GG genotype by 8.686 (*p* = 0.041). Subjects with *MET* CGA haplotype had better emotion perception by 8.748 than those without this haplotype (*p* = 0.009). However, *MET* rs41735, rs1858830 and *AKT* rs1130233 did not affect facial emotion perception (**Table 2**). In addition, each year increase in education duration increased performance of facial emotion perception by 1.86–2.06 (data not shown). Men had better facial emotion perception than women by 4.87 (*p* = 0.048) in general linear regression analysis for *MET* rs2237717. Other confounding variables did not significantly influence facial emotion perception.

**Figure 1:**
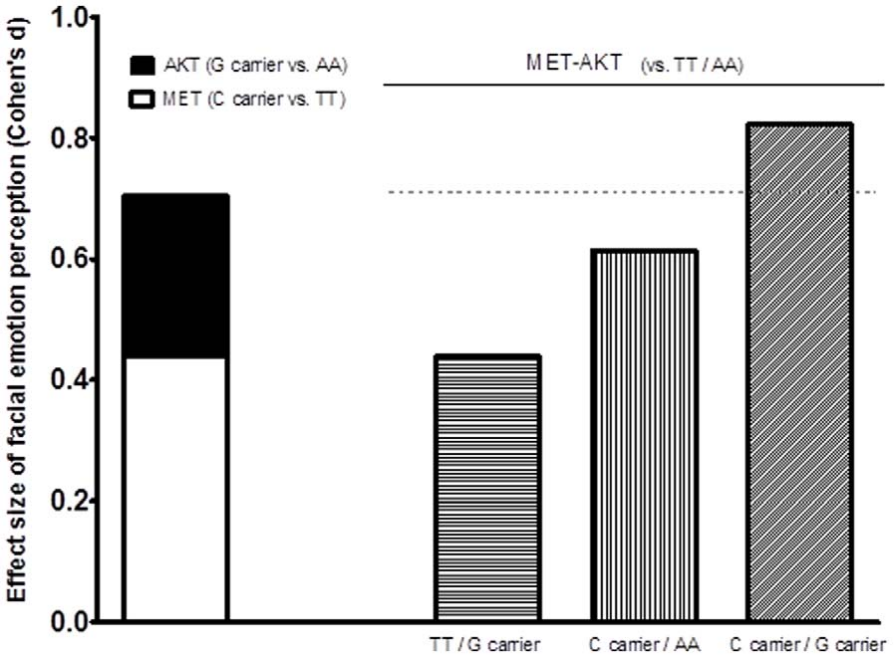
*MET* rs2237717 and *AKT* rs1130233 combined effect on facial emotion perception. Compared with TT/AA of *MET*/*AKT*, C carrier/G carrier had a Cohen's *d* (0.822), which was similar with the sum (0.704) of Cohen's *d* of *MET* rs2237717 (*d* = 0.439) and *AKT* rs1130233 (*d* = 0.265), indicating an additive effects of the two SNPs on facial emotion perception.

To explore the combined effect of *MET* and *AKT* variants, we chose *MET* rs2237717 and *AKT* rs1130233, because *MET* rs2237717 had the most significant influence on facial emotion perception in the current study and *AKT* rs1130233 had been shown to be functional in the previous studies [41], [44]. In the general linear analysis controlling age, gender, and education, the overall effect of *MET*/*AKT* on facial emotion perception was significant (*p* = 0.041). When the individuals who were C carrier and G carrier simultaneously were used as the reference group, their facial emotion perception was better than that of those with TT/AA (*p* = 0.015, **Table 2**). To inspect the influence of the combined effect of both genes on facial emotion perception, effect sizes of *MET* rs2237717, *AKT* rs1130233, and *MET*/*AKT* combination were compared (**Figure 1**).This result showed a possible additive effect of the two genes on facial emotion perception.

To evaluate the possible effect of cognitive functions on facial emotion perception, we further explored the 149 patients who also received thorough cognition assessments. Demographic data and facial emotion perception in the 149 patients **Table S2**) were similar to those in the all 182 subjects. *MET* rs2237717, *MET* CGA haplotype and *MET*/*AKT* combination still had significant effects on facial emotion perception (**Table S2**). *MET* and *AKT* variants had no significant influence on cognitive function (**Table S2**). Effects of *MET*/*AKT* variants were also analyzed by general linear regression analysis in the 149 subjects. After control for age, gender, education duration, and neurocognition, subjects with CC and CT genotypes of *MET* rs2237717 had better facial emotion perception when compared with TT genotype(*p* = 0.035, 0.037, respectively). Subjects with *MET* CGA haplotype had better emotion perception than those without this haplotype (*p* = 0.020) (details not shown). The participants who were C carrier/G carrier of *MET*/*AKT* were superior to those with TT/AA in facial emotion perception (*p* = 0.046, **Table 3**). Neurocognition did not affect facial emotion perception (**Table 3**).

**Table 3:** General linear regression analysis of the *MET*/*AKT* variants for facial emption perception in 149 subjects who also received comprehensive cognitive tests.

Parameter	Estimated coefficient	Standard error of estimated coefficient	*p*	Power
Male vs. female	7.338	3.614	0.044	
Age, y	0.186	0.194	0.338	
Education, y	1.547	0.938	0.101	
Composite cognition^*^	0.160	0.185	0.390	
*MET*-*AKT* †				
TT/AA‡	−12.890	6.394	**0.046**	**0.73**
TT/G carrier‡	−9.199	5.097	0.073	0.42
C carrier/AA‡	−3.265	4.182	0.436	0.22

MATRICS overall composite T score [58].

† *MET* rs2237717/*AKT* rs1130233.

‡ Compared with C carrier(CT+CC)/G carrier(GA+GG).

## Discussion

Sensory perception, especially facial emotion recognition, is crucial for social skill in humans [66]. To our knowledge, this study is the first one which demonstrates that genes can affect performance of facial emotion perception. The findings suggest that *MET*'s rs2237717 genotype and CGA haplotype from rs2237717, rs41735, and rs42336 of can alter facial emotion perception, and that *AKT* gene may enhance the influence of *MET* on facial emotion perception. The C allele of rs1858830 in the *MET* promoter region has been reported to be associated with autism [29], [30]; however, the current study failed to find its influence on facial emotion perception. One of the possible explanations is the different ethnicities. Previous Japanese and Chinese studies [65], [67] also did not find an association between *MET* rs1858830 and autism, but did find an association between *MET* rs38841 and 38845 and autism [65], [67]. In genetic studies, different associated variants may be reported in the same region in heterogeneous disorders such as autism and schizophrenia [68].

Burdick et al [37] found that the haplotype GCAATACA from rs38857- rs10215153- rs2237717- rs2283053- rs41735- rs41741- rs42336- rs41750 of *MET* was associated with better neurocognitive ability in healthy subjects. However, CGA haplotype in the current study did not affect cognitive function (**Table S1**). The possible reasons include differences in cognitive tests, ethnicity, and haplotypes between studies.

The A allele of *AKT* rs1130233 was reported to be associated with reduced cognitive functions [44]; the current study showed the same trend for facial emotion perception, albeit statistically insignificant (**Table S1**) (perhaps due to the modest sample size). *AKT* is also involved with emotional memory learning in the amygdala, which is a critical area in emotion perception [45]. In spite of insignificant effect of *AKT* variants on facial emotion perception, the present study revealed that *AKT* had additive effect with *MET* on facial emotion perception: the individuals who were both C carriers/G carriers of *MET*/*AKT* had better emotion perception than those with TT/AA. Therefore, this finding suggests that the *AKT* gene may modulate the *MET* effect on facial emotion perception. Such gene-gene interactions are pretty common in CNS signal pathways. One recent example is the interaction of Neuregulin-1 (NRG-1) and its receptor, ERBB4 [69], [70]. Schizophrenia patients who carried 3 *NRG1*/*ERBB4*/*AKT* risk genotypes were disproportionately worse in dorsolateral prefrontal function in the image study [69].

Consistent with a previous study [71], the current study showed that higher education level was associated with better facial emotion perception. However, *MET* genotype, haplotype and *MET/AKT* combination still exerted significant effects on facial emotion perception after control for education level.

The present study had some limitations, such as utilizing the face task of the emotion perception branch of MSCEIT, which only reflects the general ability of facial emotion perception. However, the face task of emotion perception can also be an important component of social cognition [4], [72], [73]. Second, this study focused on healthy Han-Chinese subjects. Whether the finding can be extrapolated to mentally ill patients or other races remain unknown. Third, the sample size in this study was only modest; however, the power of effects of *MET* and *MET/AKT* was medium to large. Future studies with larger samples in other ethnicities are warranted.

In summary, the results suggest that the *MET*/*AKT* cascade may play a role in facial emotion perception. Further studies in other races or in patients with mental disorders such as schizophrenia are needed.

## Supporting Information

Figure S1 Linkage disequilibrium (D′) for the *MET* SNPs was computed using Haploview 4.2.
(TIF)

Table S1 Allele frequencies of *MET* SNPs and *AKT* SNP.
(DOC)

Table S2 Demographics, facial emotion perception, composite cognition*, and *MET* SNPs and haplotypes, *AKT* SNP, and *MET*/*AKT* variants in the 149 patients who also received thorough cognitive assessments.
(DOC)
