# Supplementary figures and images for: MET and AKT Genetic Influence on Facial Emotion Perception
Source: PLoS One. 2012 Apr 27;7(4):e36143. doi: 10.1371/journal.pone.0036143 (PMC3338598; doi:10.1371/journal.pone.0036143)

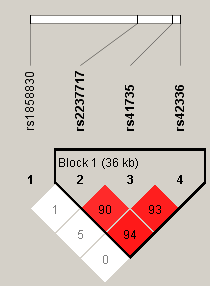

Supplement: Figure S1 — Linkage disequilibrium (D′) for the MET SNPs was computed using Haploview 4.2. (TIF) [file pone.0036143.s001.tif]
