# Supplementary material for: MET and AKT Genetic Influence on Facial Emotion Perception
Source: PLoS One. 2012 Apr 27;7(4):e36143. doi: 10.1371/journal.pone.0036143 (PMC3338598; doi:10.1371/journal.pone.0036143)
Supplement: Table S1 — Allele frequencies of MET SNPs and AKT SNP. (DOC) [file pone.0036143.s002.doc]

**Table S1** Allele frequencies of *MET* SNPs and *AKT* SNP

|  | | | | Allele frequency |
| --- | --- | --- | --- | --- |
| ***MET****-***rs2237717** | C/T |  | 0.53/0.47 | |
| ***MET*- rs41735** | G/A |  | 0.55/0.45 | |
| ***MET*- rs42336** | A/G |  | 0.54/0.46 | |
| ***MET*- rs1858830** | G/C |  | 0.67/0.33 | |
| ***AKT*- rs1130233** | A/G |  | 0.55/0.45 | |
