# Supplementary material for: MET and AKT Genetic Influence on Facial Emotion Perception
Source: PLoS One. 2012 Apr 27;7(4):e36143. doi: 10.1371/journal.pone.0036143 (PMC3338598; doi:10.1371/journal.pone.0036143)
Supplement: Table S2 — Demographics, facial emotion perception, composite cognition*, and MET SNPs and haplotypes, AKT SNP, and MET/AKT variants in the 149 patients who also received thorough cognitive assessments. (DOC) [file pone.0036143.s003.doc]

**Table S2** Demographics, facial emotion perception, composite cognition*, and *MET* SNPs and haplotypes, *AKT* SNP*,* and *MET*/*AKT* variants in the 149 patients who also received thorough cognitive assessments

|  | Male/Female | Age (SD) | Education (SD) | Facial emotion perception (SD) | Composite cognition*(SD) |
| --- | --- | --- | --- | --- | --- |
| ***MET****-***rs2237717** |  |  |  |  |  |
| CC | 18/28 | 30.7(10.3) | 14.5(2.3) | 92.3(22.3) | 50.1(10.6) |
| CT | 30/41 | 31.9(9.4) | 15.1(1.7) | 93.1(19.4) | 50.1(10.4) |
| TT | 15/17 | 30.7(10.1) | 14.2(2.4) | 82.4(21.1) | 49.6(8.2) |
| *p*^†^ | 0.793 | 0.745 | 0.121 | **0.046** | 0.968 |
| ***MET*- rs41735** |  |  |  |  |  |
| GG | 18/28 | 30.6 (10.4) | 14.5(2.3) | 92.3(22.3) | 49.8(10.7) |
| GA | 33/40 | 320(9.2) | 14.9(1.7) | 91.8(20.3) | 50.3(10.4) |
| AA | 12/18 | 30.7(9.8) | 14.4(2.6) | 84.9(21.0) | 49.6(8.0) |
| *p*^†^ | 0.776 | 0.730 | 0.391 | 0.253 | 0.936 |
| ***MET*- rs42336** |  |  |  |  |  |
| GG | 14/18 | 30.8(10.1) | 14.3(2.6) | 83.8(21.1) | 49.3(8.0) |
| AG | 32/41 | 31.8(9.3) | 15.0(1.7) | 92.6(19.8) | 50.2(10.4) |
| AA | 17/27 | 30.8(10.5) | 14.4(2.2) | 92.2(22.1) | 50.2(10.7) |
| *p*^†^ | 0.844 | 0.832 | 0.142 | 0.120 | 0.906 |
| ***MET*- rs1858830** |  |  |  |  |  |
| GG | 31/33 | 31.8(10.2) | 14.6(1.9) | 90.2(21.1) | 49.3(9.6) |
| GC | 25/48 | 31.2(9.6) | 14.7(2.3) | 91.3(21.9) | 50.0(10.5) |
| CC | 7/5 | 29.3(9.0) | 14.8(1.8) | 88.3(17.9) | 54.0(8.3) |
| *p** | 0.123 | 0.715 | 0.947 | 0.889 | 0.325 |
| ***MET*- CGA** |  |  |  |  |  |
| carrier | 47/68 | 31.5(9.8) | 14.8(2.0) | 92.9(20.6) | 50.2(10.5) |
| Non carrier^‡^ | 15/17 | 30.7(10.1) | 14.2(2.4) | 82.4(21.9) | 49.6(8.2) |
| *p*^†^ | 0.543 | 0.683 | 0.150 | 0.**014** | 0.776 |
| ***AKT*- rs1130233** |  |  |  |  |  |
| AA | 16/30 | 30.1(9.8) | 14.4(2.6) | 86.4(21.2) | 48.6(10.8) |
| AG | 33/38 | 32.6(10.1) | 14.7(1.9) | 91.9(23.7) | 49.8(10.1) |
| GG | 14/18 | 29.8(8.9) | 15.1(1.8) | 93.7(113.3) | 52.4(8.3) |
| *p*^†^ | 0.449 | 0.223 | 0.407 | 0.250 | 0.246 |
| ***MET-AKT***^§^ |  |  |  |  |  |
| TT/AA | 4/8 | 30.5(10.4) | 13.8(3.3) | 78.1 (19.6) | 47.7(9.5) |
| TT/G carrier | 11/9 | 30.8(10.2) | 14.5(1.8) | 85.4(23.3) | 50.7(7.4) |
| C carrier/AA | 12/22 | 30.0(9.8) | 14.7(2.3) | 89.3(21.3) | 48.9(11.3) |
| C carrier/G carrier | 36/47 | 32.1(9.7) | 14.9(1.9) | 94.2(20.1) | 50.6(10.1) |
| *p*^†^ | 0.486 | 0.738 | 0.333 | **0.041** | 0.688 |

* MATRICS overall composite T score [[58]](#_ENREF_56)

^†^Chi-Square test for gender comparison; ANOVA for other items

^‡^ Non-carriers of CGA included those with TAG or CAG

^§^ *MET* rs2237717/*AKT* rs1130233
